# A dataset of DIMACS formulas derived from Kconfig models for research on highly configurable software

**DOI:** 10.1016/j.dib.2026.112747

**Published:** 2026-04-03

**Authors:** Ruben Heradio, Cristina Cerrada, Ismael Abad, Ernesto Aranda, Juan Jose Escribano, David Fernandez-Amoros

**Affiliations:** Universidad Nacional de Educación a Distancia (UNED), Calle Juan del Rosal 16, Madrid, 28040, Spain.

**Keywords:** Software product line, Configurable system, Linux, SAT solver, Knowledge compilation

## Abstract

Translating Kconfig models into DIMACS-encoded Boolean formulas enables automated reasoning over configurable software systems. However, a complete translation encompassing the entire Kconfig language is still lacking. KconfigReader and KMax are two of the most widely adopted translators, yet no large-scale dataset has previously been available to systematically compare their outputs, evaluate the implications of their translations for reasoning tasks, or benchmark reasoning algorithms on extensive DIMACS files derived from Kconfig models.

The dataset presented here consists of 5,476 DIMACS files, representing the KconfigReader and KMax encodings of 2,738 Kconfig models from nine open-source systems (axTLS, Buildroot, BusyBox, EmbToolkit, Freetz-NG, L4Re, the Linux kernel across 43 architectures, Toybox, and uClibc), covering multiple releases and a wide range of complexity. This dual-translation design supports three primary research tasks: (i) comparing KconfigReader and KMax to identify translation gaps; (ii) benchmarking complex reasoning operations, such as backbone solving, model counting, and configuration sampling across varying levels of complexity; and (iii) tracking the evolution of Kconfig across software releases.

Specifications Table

**Table:** 

Subject	Computer Sciences
Specific subject area	Highly Configurable Software
Type of data	Raw
Data collection	The dataset was generated automatically using torte, a declarative experimentation platform that runs each pipeline stage inside Docker containers for reproducibility. For every tagged release of the nine target systems, torte cloned the source repository, invoked KconfigReader and KMax to extract the Kconfig model, transformed each model to SMT-LIB 2 format with FeatJAR, and converted it to DIMACS using the Z3 solver. The inclusion criterion was all publicly tagged versions in each system’s official repository. No manual intervention or postprocessing was applied.
Data source location	ETSI Informática de la Universidad Nacional de Educación a Distancia (UNED), Calle Juan del Rosal 16, 28040 Madrid, Spain
Data accessibility	Repository name: Raw DIMACS Formulas Derived from Kconfig Models for Research on Highly Configurable Software DOI: 10.5281/zenodo.19259177 Direct URL to data: https://doi.org/10.5281/zenodo.19259177
Related research article	None

## 1 Value of the Data

• The dataset contains (i) 2,738×2 = 5,476 DIMACS files (29.5 GB) from nine open-source systems, each one translated by KconfigReader and KMax; (ii) summary.csv records per-formula metrics; (iii) features_and_variable_ids/ provides 2,738 CSV tables mapping shared feature names to their variable IDs in each translator's DIMACS file; and (iv) replication.sh regenerates all files from source.
• The dual translations of the same 2,738 models enable direct comparison of KconfigReader and KMax, revealing which Kconfig constructs (tristate options, non-Boolean configs, order-dependent selections) cause the greatest divergence.
• The DIMACS files form a ready-to-use benchmark for SAT solving, #SAT counting, backbone computation, configuration sampling, and knowledge compilation, spanning from embedded models with 22 variables to Linux kernel formulas with up to 73,719 variables.
• Coverage of multiple tagged releases enables longitudinal studies of Kconfig evolution, tracking how options, constraints, and core/dead features change over time. The 43 Linux kernel architectures additionally support cross-platform variability analysis.
• The formulas support automated reasoning for software quality and security: dead-feature detection, configuration-space sampling for test coverage, and dependency analysis for safety-critical configurations.
• The paired KconfigReader/KMax encodings of 2,738 identical models provide a reference benchmark for validating future Kconfig-to-Boolean translators: a new tool can be evaluated by checking whether its output preserves the satisfiability structure and key formula properties (e.g., core and dead features) of the existing encodings.

## 2 Background

Variability in software systems is frequently specified using dedicated languages such as Kconfig, which models configuration options and their constraints. These models become highly complex in large-scale systems; for instance, the Linux kernel 6.8.4 model comprises 18,267 options distributed across 1,682 files [1]. Effectively managing this complexity is essential for tasks such as defining valid configurations, identifying essential or unused options [2,3], refactoring models [4], optimizing performance [5], and assessing security vulnerabilities [6]. The primary method for addressing these tasks involves translating Kconfig models into Boolean formulas, which are subsequently processed by logic engines [7]. However, this approach encounters significant challenges. Translation tools remain incomplete [[8], [9], [10], [11], [12]] due to Kconfig ambiguous semantics, its use of non-Boolean options, and its imperative characteristics, where the order of options and constraints influences the resulting configurations. Scalability also presents difficulties for several critical tasks, such as backbone detection and configuration sampling [1,5,13,14]. This dataset supports research on these problems by providing Boolean formulas derived from various open-source systems, encoded in the DIMACS standard format and generated by two state-of-the-art translators: KconfigReader [15] and KMax [16]. The dataset complements the resource published in [17], which is limited to Linux and whose DIMACS files are preprocessed using backbone simplification [18]. In contrast, our dataset of raw DIMACS files enables researchers to evaluate translation limitations and analyze the scalability of tasks across a broader range of Kconfig systems.

## 3 Data Description

**Fig. 1:**
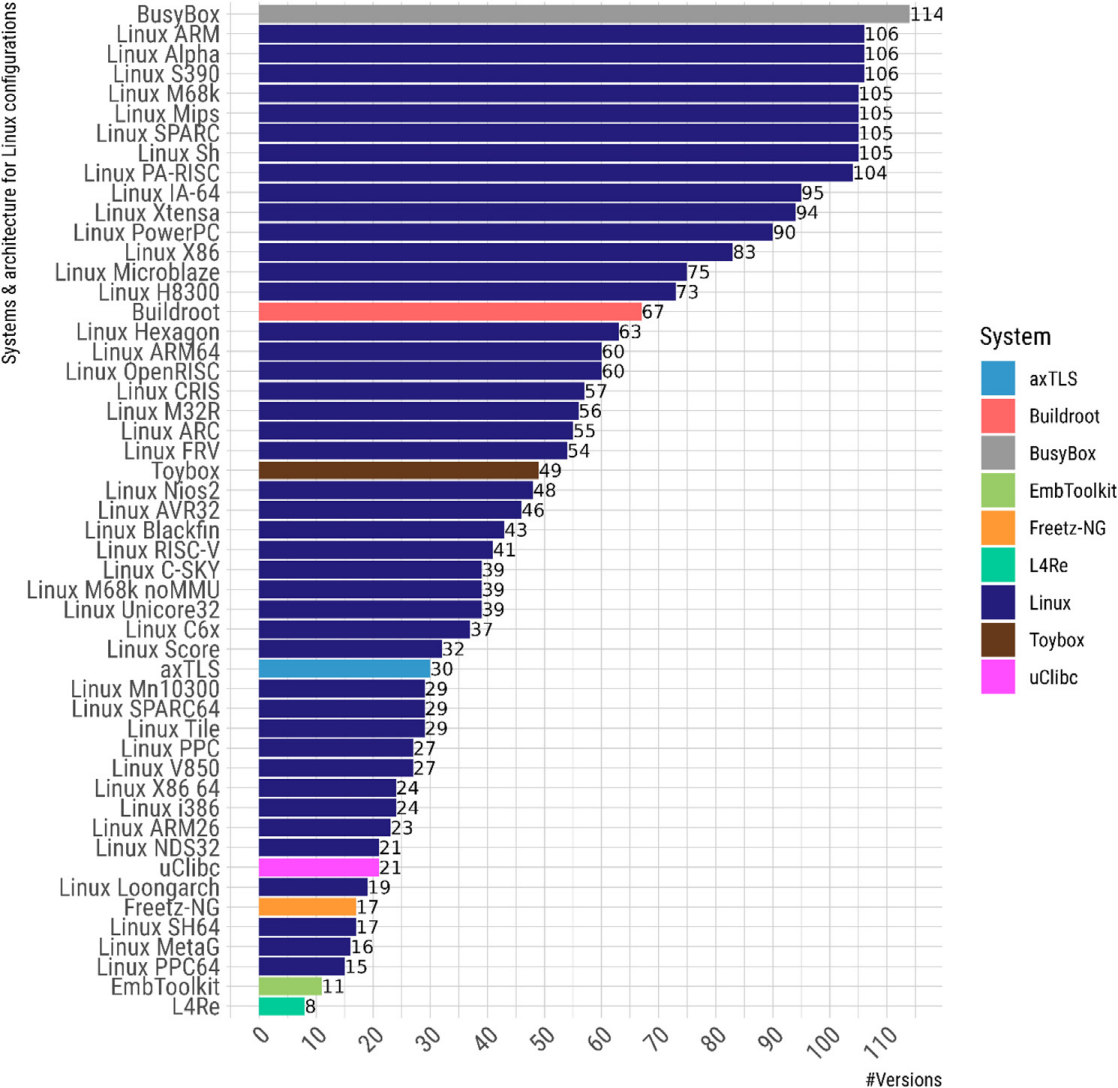
Distribution of system versions, distinguishing among architectures for Linux.

The dataset comprises the DIMACS encodings, obtained with KconfigReader and KMax, for 2,738 Kconfig models across multiple versions of 9 open-source systems: axTLS, Buildroot, BusyBox, EmbToolkit, Freetz-NG, L4Re, Linux kernel, Toybox, and uClibc. For the Linux kernel, the dataset also includes base configurations for 43 architectures, including ARM, M68k, and x86. Fig. 1 summarizes the number of versions in the dataset per system and Linux architecture.

This section is organized as follows: First, we present the syntax and semantics of DIMACS files, with a focus on their use in encoding Kconfig models; Then, we examine the complexity of the DIMACS files within the dataset, highlighting implications for future research on algorithm scalability; Finally, we describe the repository structure and the summary.csv file, which enumerates the main properties of the DIMACS files.

### 3.1 DIMACS files for Kconfig models

As an illustrative example, Listing 1 provides an excerpt from the Linux 2.6.0 Kconfig model, which contains 3,678 configuration options distributed across 202 files. Specifically, the code references 12 options for the Advanced Configuration and Power Interface (ACPI), which facilitates communication between the operating system and the computer’s firmware to manage power, support hibernation, enable device auto-configuration, and promote energy efficiency. Each configurable option is represented as a Boolean config, which can be enabled or disabled. The listing demonstrates the following constraint types:

• Dependencies and conflicts are indicated by the keyword “depends on”; for example, enabling ACPI_NUMA requires ACPI_INTERPRETER to be enabled (Line 9) and X86_64 to be disabled (Line 11).
• Default values are assigned based on an “if” condition; for instance, NUMA is enabled whenever IA64_GENERIC, IA64_DIG, or IA64_HP_ZX1 are enabled (Line 25).
• Choices are defined using the “choice...endchoice” construct; for example, each configuration must include exactly one of IA64_GENERIC, IA64_DIG, IA64_HP_ZX1, or IA64_SGI_SN2 (Lines 17-22).

Translators, such as KconfigReader and KMax, convert Kconfig models into DIMACS files, which encode Boolean formulas in Conjunctive Normal Form (CNF) [19]. A CNF is a conjunction of one or more clauses, where each clause is a disjunction of literals, which can be either a variable or a negated variable. For example, the following CNF formula φ encodes Listing 1:

**Figure:**
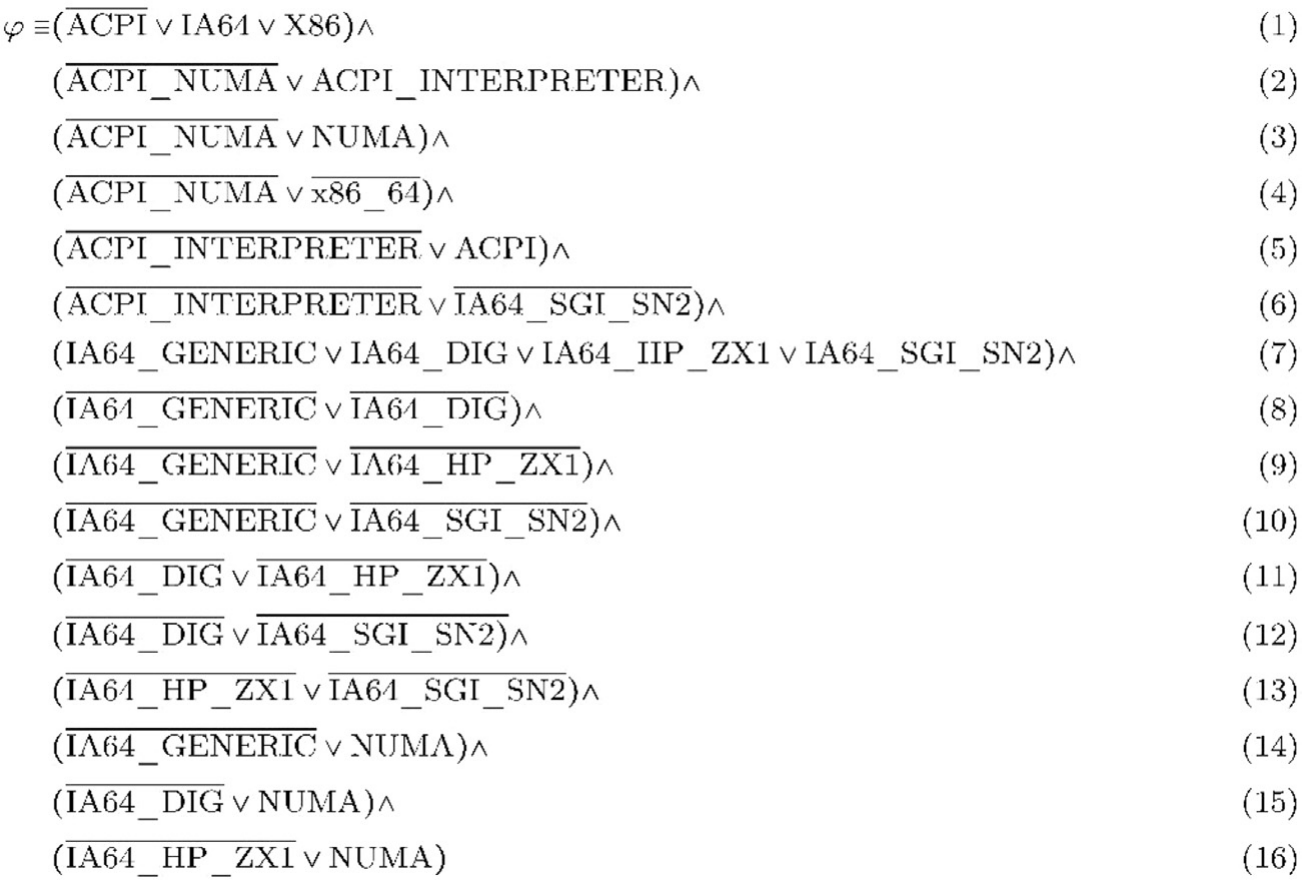

φ has 11 variables, 16 clauses, and its 1st clause has 3 literals: ACPI, IA64, and X86.

**Listing 1:**
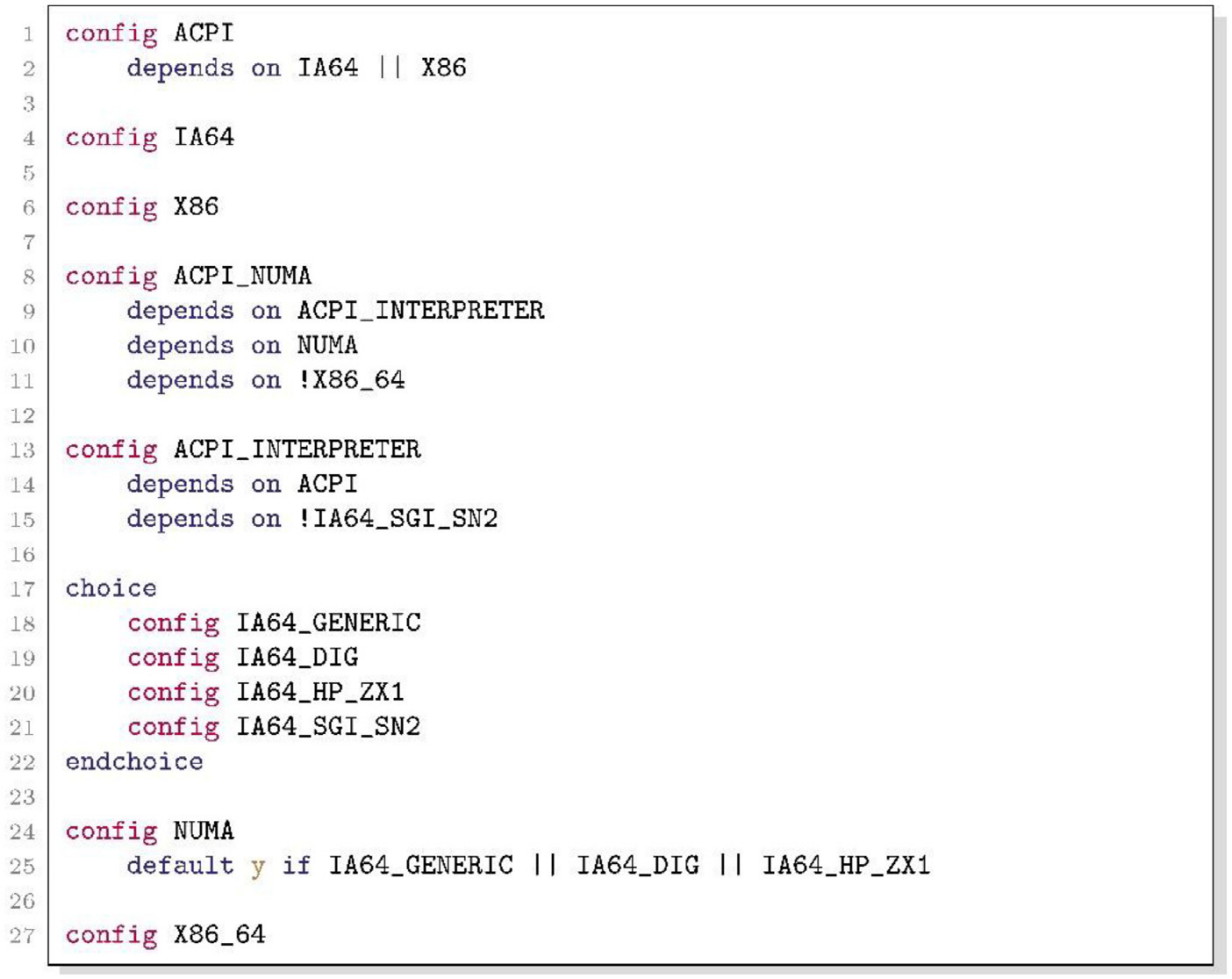
An excerpt from the variability model of Linux 2.6.0

Listing 2 shows the corresponding DIMACS encoding. The file starts with comments, marked by the letter “c”, that assign natural numbers to configurable options in Lines 1-11. Line 12 contains “p cnf n c” indicating the DIMACS file uses the CNF format, where n is the number of variables in φ and c is the number of clauses. Lines 13-28 encode the clauses using non-zero numbers from −n to n. Zeros serve as end-of-clause markers. Positive numbers denote affirmed literals; negative numbers denote their negations. For example, Line 13 encodes the 1st clause ACPI ∨ IA64 ∨ X86.

**Listing 2:**
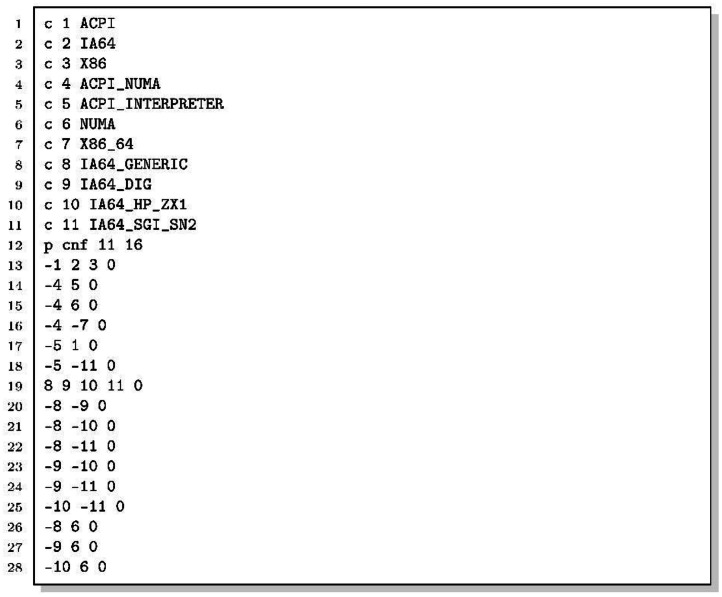
DIMACS file encoding the Boolean formula φ presented in Listing 1

The simplified example in Listing 2 uses only Boolean options and is small enough that no Tseitin auxiliary variables are introduced. In the actual DIMACS files of the dataset, which encode real-world Kconfig models, the comment block additionally contains entries for Tseitin variables introduced by Z3′s tseitin-cnf tactic [21,22]. These variables do not correspond to any Kconfig option; they are purely structural, representing sub-formula nodes that allow CNF conversion in linear rather than exponential size. They are consistently named k!N (where N is a non-negative integer, e.g., k!0, k!1, k!2), and they always appear at the end of the comment block, immediately before the p cnf header line. Researchers can therefore reliably distinguish Tseitin variables from Kconfig-derived variables by checking whether a variable name starts with the prefix k!.

### 3.2 Complexity of the DIMACS files in the dataset

**Fig. 2:**
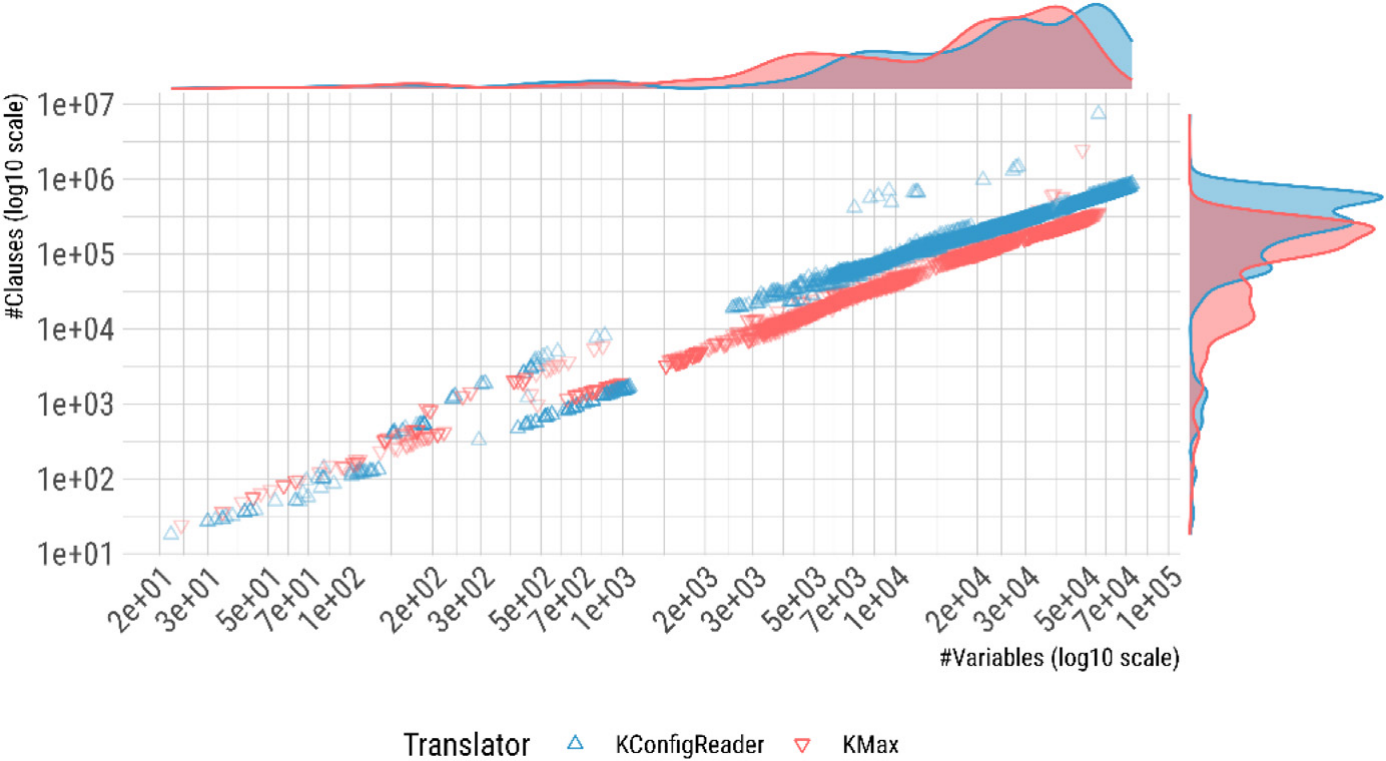
Scatter plot illustrating the complexity of the formulas in the dataset. Each point represents a formula, with the x- and y-coordinates corresponding to the number of variables and clauses, respectively. The density diagrams at the top and right display the marginal distributions of the number of clauses and variables, respectively.

Fig. 2 shows the complexity of the formulas in the dataset, measured by their number of variables and clauses. Note that KconfigReader translations are generally simpler than KMax translations for smaller models but become more complex for the largest models. A preliminary hypothesis for this divergence is that KconfigReader's encoding grows faster than KMax's as the number of tristate and integer/hexadecimal options in a model increases. KconfigReader introduces, unconditionally, two Boolean variables per tristate option (one for state y, one for m) and one Boolean variable per enumerated value for each integer or hexadecimal option [15]. In large systems such as the Linux kernel, which has thousands of tristate options and hundreds of integer/hexadecimal options with multiple enumerated values, the cumulative effect of these encodings, together with the mutual-exclusion constraints between value variables, substantially inflates the variable and clause counts. KMax, by contrast, collapses each non-Boolean option to a single Boolean availability variable and introduces the extra tristate variables only when they appear in constraint expressions [16]. For small embedded systems with few such options, KMax's guard variables (__VISIBILITY__CONFIG_*) add proportionally more overhead, which explains why KconfigReader formulas are smaller for those models. As models grow larger and more feature-rich, the balance tips in the other direction.

**Fig. 3:**
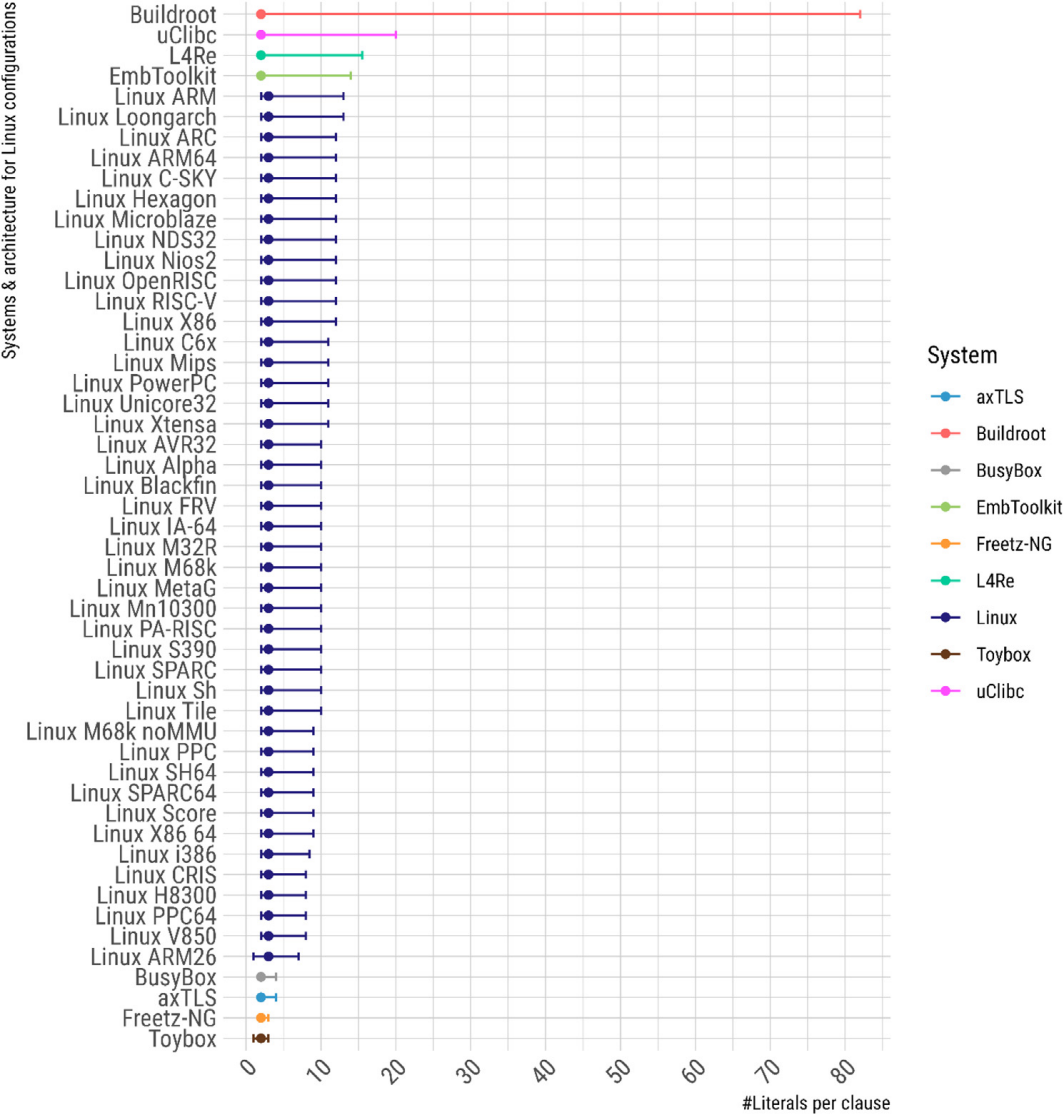
Bars indicating the 95 % coverage interval for the number of literals per clause; the central point denotes the median, which oscillates between 2 and 3 literals per clause.

When all clauses in a CNF contain at most two literals, the satisfiability (SAT) problem, which is central for most operations on formulas, is referred to as 2-SAT and can be solved in polynomial time (see Section 1.19 in [19]). In contrast, the presence of clauses with more than two literals renders the problem NP-complete. Fig. 3 shows the SAT-hardness of the formulas by depicting, for all system versions, the median of (i) the median number of literals per clause (i.e., a double median), and (ii) the 95 % coverage interval for the number of literals per clause.

### 3.3 Organization of the repository

**Fig. 4:**
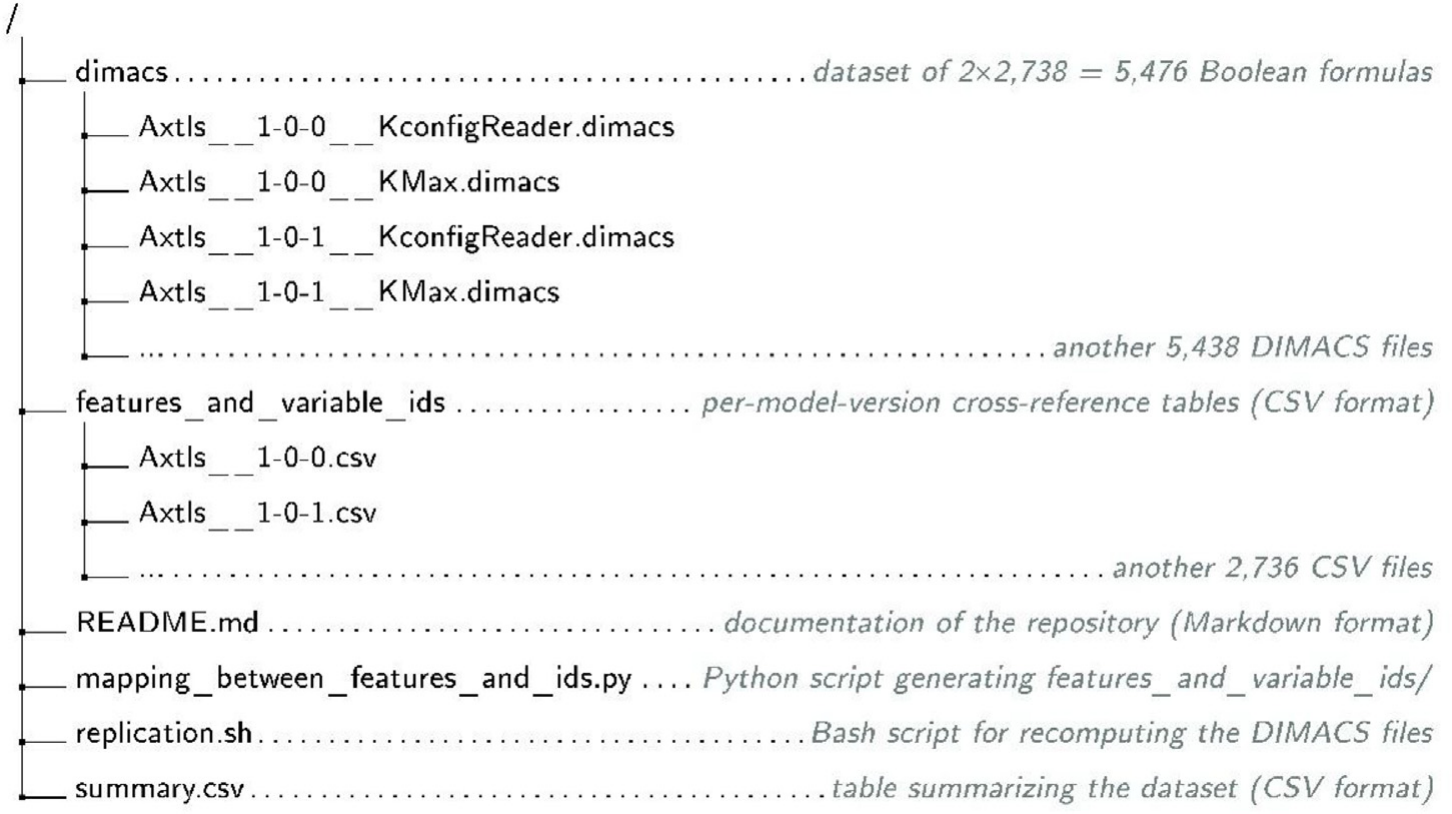
Organization of the repository.

Fig. 4 shows the organization of the repository.

1. The dimacs folder contains the 5,476 DIMACS files (see Section 3.1): two per Kconfig model, one from KconfigReader and one from KMax.
2. The features_and_variable_ids folder contains 2,738 CSV files, one per model version. Each file lists every Kconfig feature that appears in both the KconfigReader and KMax translations of that model, together with the integer variable ID assigned to it in each DIMACS file. The three columns are: Feature, KConfigReaderId, and KMaxId. Tseitin auxiliary variables (k!N) are excluded because, although they share a naming convention, they represent unrelated subformula nodes in the two files and carry no shared semantics.
3. README.md summarizes the repository’s content.
4. mapping_between_features_and_ids.py is a Python script that generates the features_and_variable_ids/ tables from the DIMACS files. It is invoked automatically at the end of replication.sh and can also be run independently.
5. replication.sh is a Bash script that regenerates the DIMACS files from the systems’ online repositories (see Section 4).
6. summary.csv is a CSV file with one row per DIMACS file and the following columns:
  • Name: Three-field identifier separated by “ ”: (i) system name (with architecture appended for Linux), (ii) version (dots replaced by “-” to avoid file-extension ambiguity), and (iii) translator. Example: LinuxArm64 6-5 KMax.dimacs.
  • NumberOfVariables: Number of variables.
  • NumberOfClauses: Number of clauses.
  • NumberOfCoreFeatures: Variables that must be true in every satisfying assignment.
  • NumberOfDeadFeatures: Variables that must be false in every satisfying assignment.
  • MedianLiteralsPerClause: Median number of literals per clause.
  • Inf95CovIntLitsPerClause: Lower bound of the 95 % coverage interval for literals per clause.
  • Sup95CovIntLitsPerClause: Upper bound of the 95 % coverage interval for literals per clause.
  • Version: System version (dots, not “-”).
  • ToolToGetTheFormula: Translator used, KconfigReader or KMax.

## 4 Experimental Design, Materials and Methods

Torte [17] is a declarative experimentation platform that wraps every tool in a Docker (or Podman) container, pinning compiler, library, and solver versions so that results are identical across machines and over time (Table 1). The dataset repository includes the self-contained replication script replication.sh (Listing 3); running it on a machine with Docker installed regenerates all 5,476 DIMACS files without manual intervention.

**Table 1:** Docker containers, tool versions, and their role in the pipeline.

Container	Base image	Key software	Stage	Purpose
torte_util	Ubuntu 22.04	Git, GNU parallel	1	Repository cloning
torte_kconfigreader	Ubuntu 14.04	OpenJDK 7, sbt 0.13.6, GCC 9	2	Kconfig extraction
torte_kclause	Ubuntu 22.04	Python 3, KMax version 414adb9	3	Kconfig extraction
torte_featjar	Ubuntu 22.04	FeatJAR version 3fc8d66, OpenJDK 17, Gradle 9.1.0	4	Model-to-SMT transformation
torte_z3	Ubuntu 22.04	Python 3, Z3 4.11.2	5	SMT-to-DIMACS conversion

**Listing 3:**
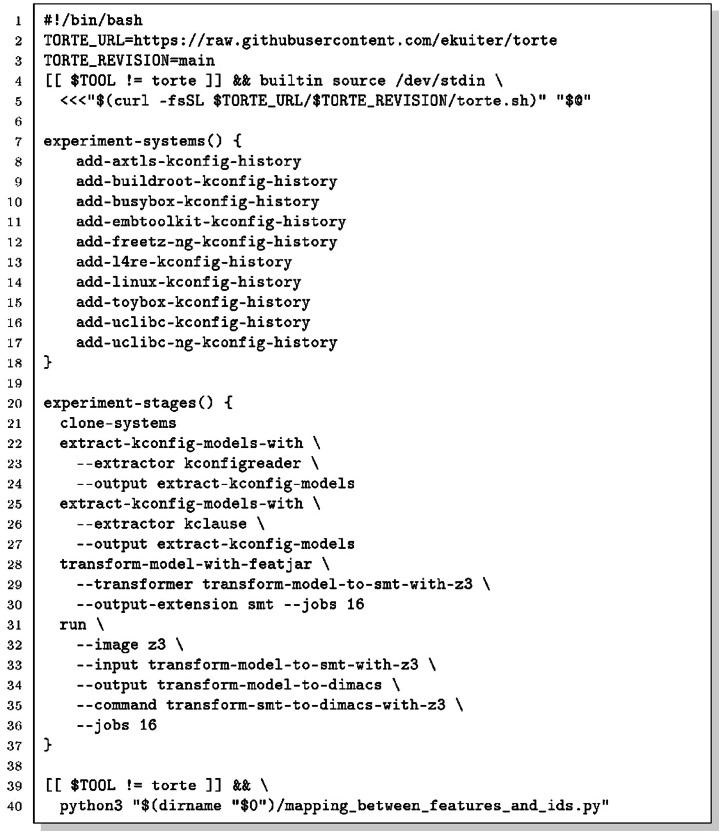
Bash script that runs torte to generate the DIMACS files from the systems online repositories.

Lines 2-5 bootstrap the torte framework at runtime: curl downloads the entry-point script torte.sh from GitHub (at the revision specified by TORTE_ REVISION), and the shell sources it via stdin, making all torte commands available in the current process. The guard [[ $TOOL != torte]experiment-stages (Lines 20–37) declares five pipeline stages. Lines 39-40 run mapping_between_features_and_ids.py after the pipeline completes to generate the cross-reference CSV tables in features_and_variable_ids/; the guard [[ $TOOL != torte]] ensures this step executes only during the initial user invocation, not when torte re-invokes the script internally.

1. Repository cloning (Line 21). clone-systems clones each registered repository and checks out every tagged version.
2. KconfigReader extraction (Lines 22–24). KconfigReader [15] compiles the dumpconf utility against the system’s Kconfig C library, serialises the model to XML, and produces a propositional Boolean formula in its internal .model format. These files contain arbitrary Boolean expressions (AND, OR, NOT, implications, biconditionals) that are not yet in CNF. Non-Boolean Kconfig types are encoded as follows. Tristate options x are split into two mutually exclusive Boolean variables x_y and x_m representing states 'y' and 'm'; state 'n' is modelled by NOT(x_y) AND NOT(x_m). This pair is always introduced regardless of whether the tristate values are referenced in constraints. Integer and hexadecimal options receive one Boolean variable per value explicitly mentioned in the model's default directives or constraint literals, together with a _n variable for 'unset'; values absent from the model are not represented. String options are handled identically to integer options: one Boolean variable per explicitly-referenced value. The variable-name suffixes _0, _1, _nonempty, and _n visible in the comment block of KconfigReader DIMACS files reflect this encoding.
3. KMax extraction (Lines 25–27). The kclause component of KMax [16] parses the Kconfig files (kextract) and converts them into propositional clauses (kclause). Both KconfigReader and KMax write to the same staging directory; torte distinguishes them by embedding the translator name in each filename. KMax handles non-Boolean Kconfig types differently from KconfigReader. When a tristate option appears in a constraint expression, two Boolean variables are introduced (x==y and x==m), linked to the base variable x via a biimplication; tristate choices are approximated as Boolean mutex choices (preventing multiple members from being set to 'm'). Integer, hexadecimal, and string options are each reduced to a single Boolean availability variable indicating whether the option has any value; the actual value is discarded. Non-visible non-Boolean options (those without a user-facing prompt) are omitted from the formula entirely. The __VISIBILITY__CONFIG_ variables present in KMax DIMACS files are guard variables that model the conditional visibility of options.
4. SMT transformation (Lines 28–30). FeatJAR reads the propositional models from both extractors and converts them to a uniform SMT-LIB 2 representation, which serves as the common intermediate format in the pipeline.
5. DIMACS conversion (Lines 31–36). The Z3 SMT solver [20], version 4.11.2, converts each SMT-LIB 2 file to DIMACS CNF by applying three tactics in sequence: first, simplify performs constant folding and algebraic simplifications; then, elim-and rewrites the formula using only ∨ and ¬; finally, tseitin-cnf applies the Tseitin transformation [21,22], which introduces auxiliary variables to produce a CNF of size O(n) instead of incurring the exponential blowup of a naive CNF generation based on repeatedly applying the distributive property of disjunction over conjunction. Tseitin auxiliary variables are documented in the DIMACS comment block under names of the form k!N (e.g., k!0, k!1), positioned at the end of the comment block immediately before the p cnf header line. They do not map back to any Kconfig option and can be identified by the k! prefix.
6. Cross-reference table generation (Lines 39–40). After the torte pipeline completes, mapping_between_features_and_ids.py generates one CSV file per model version in features_and_variable_ids/, listing every Kconfig feature that appears in both translations together with its integer variable ID in each DIMACS file.

Table 2 contrasts the variable sets produced by the two translators for a minimal tristate example where config BAR depends specifically on FOO=m. It illustrates that not only the count but also the naming of variables differs between tools, even when encoding the same concept (the module state of FOO), KconfigReader calls it CONFIG_FOO_m while KMax calls it CONFIG_FOO==m.

**Table 2:** Variables introduced by KconfigReader and KMax for a minimal Kconfig fragment: config FOO (tristate) and config BAR (tristate, depends on FOO=m).

Kconfig option	KconfigReader variables	KMax variables
config FOO (tristate)	CONFIG_FOO_y, CONFIG_FOO_m	CONFIG_FOO, CONFIG_FOO==m
config BAR (tristate) depends on FOO=m	CONFIG_BAR_y, CONFIG_BAR_m	CONFIG_BAR
Encoded dependency	(BAR_y OR BAR_m) → FOO_m	BAR → FOO==m
Variable count for these two options	4	3

Because both KconfigReader and KMax produce propositional formulas in different internal formats, the pipeline channels all models through the same FeatJAR+Z3 transformation. This separation of concerns avoids duplicating CNF-conversion logic in each extractor and ensures that any differences between the resulting DIMACS files are attributable solely to the extraction step, enabling a fair comparison between translators.

## Limitations

The data collection process is fully automated by torte, so no limitations arise from the collection procedure itself. The dataset potential limitations are inherent to KconfigReader and KMax. Despite numerous attempts to translate Kconfig into Boolean logic [[8], [9], [10],^15^,^11^,^12^], none is complete. Three core difficulties explain this. First, Kconfig semantics lack a complete formal definition; the official reference is the implementation itself, and although She and Berger [23] formalised some core aspects, many remain ambiguous. Second, Kconfig includes non-Boolean options (tristate, integer, hexadecimal, and string configs) whose translation to propositional logic requires approximation. Third, Kconfig is not fully declarative: its imperative configuration mechanism means that the order in which options are selected can alter the resulting configuration values, making a complete and sound translation difficult to guarantee. Precisely for these reasons, the dataset presented here is valuable: by providing DIMACS encodings of 2,738 real-world Kconfig models generated by both translators, it enables researchers to study and compare their strengths and weaknesses at scale, and to advance towards more complete and better-validated Kconfig-to-DIMACS translators.

## Ethics Statement

The authors confirm that we have read and comply with the ethical requirements for publication in Data in Brief, as outlined in Elsevier’s policies and ethics. Furthermore, the present work does not involve human subjects, animal experiments, or data collected from social media platforms.

## CRediT Author Statement

**Ruben Heradio:** Conceptualization, Data curation, Formal analysis, Funding acquisition, Investigation, Methodology, Project administration, Resources, Software, Supervision, Validation, Visualization, Writing - original draft, Writing - review & editing. **Cristina Cerrada:** Data curation, Investigation, Validation, Writing - review & editing. **Ismael Abad:** Data curation, Investigation, Methodology, Software, Validation, Writing - review & editing. **Ernesto Aranda-Escolastico:** Conceptualization, Data curation, Formal analysis, Methodology, Validation, Writing - review & editing. **Juan Jose Escribano:** Data curation, Investigation, Validation, Writing - review & editing. **David Fernandez-Amoros:** Conceptualization, Funding acquisition, Project administration, Resources, Supervision, Validation, Writing - review & editing.

## Data Availability

zenodoRaw DIMACS Formulas Derived from Kconfig Models for Research on Highly Configurable Software (Original data).
